# The Role of Integrative Therapies in the Management of Trauma-related Disorders Amongst Refugees

**DOI:** 10.1007/s10903-025-01770-2

**Published:** 2025-10-08

**Authors:** Tahmineh Salehi, Prem Chopra, Heidi Wegner, Jenny Adams, Josef Szwarc

**Affiliations:** 1 Victorian Foundation for Survivors of Torture (Foundation House), 4 Gardiner Street, Brunswick, Victoria 3056 Australia; 2 Melbourne School of Population and Global Health, The University of Melbourne, 207 Bouverie Street, Carlton, Victoria 3053 Australia

**Keywords:** Trauma-related disorders, Post-traumatic stress disorder, Integrative therapies, Complementary therapies, Refugees

## Abstract

**Background:**

It is well recognised that there is a high prevalence of trauma-related disorders amongst people of refugee background. Integrative approaches that utilise complementary therapies in combination with conventional Western treatments including psychological therapy and pharmacotherapy are consistent with culturally responsive care.

**Aim:**

To explore the evidence base for complementary therapies in the management of trauma-related disorders amongst refugees and summarise the evidence base for integrative approaches of relevance to the management of trauma-related disorders in this population.

**Methods:**

A narrative review was performed using the electronic database Medline (via PubMed). Additional articles were sourced using the snowball search method.

**Results:**

81 articles were identified in a preliminary search, of which 22 articles were considered eligible for inclusion in the review. A broad range of complementary therapies have demonstrated some evidence in the management of trauma-related disorders amongst refugees including herbal medicine, dance/movement therapy, art therapy, music therapy, yoga and meditation, Qigong and Tai chi, acupuncture and neurofeedback.

**Conclusion:**

There is an opportunity for health professionals to provide holistic and culturally responsive care to patients of refugee background in the management of trauma-related disorders by integrating complementary therapies with conventional treatments. Further research in this field is warranted to explore the role of specific modalities of complementary therapies.

## Introduction

### Definition of Trauma-related Disorders

Exposure to trauma in various forms may lead to significant emotional distress and impairment in functioning. Trauma-related disorders encompass a variety of specific conditions that can manifest as a result of exposure to traumatic experiences. Furthermore, as a consequence of exposure to trauma, many individuals experience depressive disorders, anxiety disorders and substance use disorders [1].

Various classification systems have been used to categorise specific trauma-related disorders. As per the American Psychiatric Association’s Diagnostic and Statistical Manual of Mental Disorders, Fifth Edition (DSM-5 TR), trauma-related disorders include Acute Stress Disorder and Post-traumatic stress disorder (PTSD). A diagnosis of Acute Stress Disorder is considered when post-traumatic stress symptoms persist for more than two days, while a diagnosis of PTSD is made if symptoms endure for over a month. As per the DSM-5 TR criteria, the diagnosis of PTSD encompasses intrusion symptoms associated with the traumatic event, persistent avoidance of stimuli associated with the event, negative alternations in cognition and mood, and alteration in arousal and reactivity [2]. According to ICD-11 criteria, PTSD encompasses three core symptom clusters (re-experiencing, avoidance of traumatic reminders, and hyperarousal) [3]. ICD-11 also defines Complex PTSD (CPTSD) that may affect a subset of individuals particularly those who have endured prolonged, repeated and interpersonal traumas, who may exhibit a distinct set of features alongside the core symptoms of PTSD. The ICD-11 diagnosis of CPTSD consists of six symptom clusters: the three PTSD criteria as well as three disturbances of self-organisation symptoms (emotional dysregulation, interpersonal difficulties, and negative self-concept) [3].

### Management of Trauma-related Disorders

Various treatment guidelines in Western countries have been developed to provide guidance for clinicians in the management of trauma-related disorders. These include the ‘Guidelines for the Management of Conditions Specifically Related to Stress’ (World Health Organisation) [4], ‘Clinical Practice Guideline for the Treatment of PTSD’ (American Psychological Association) [5], ‘VA/DoD Clinical Practice Guidelines: Management of Posttraumatic Stress Disorder and Acute Stress Disorder’ (US Veterans Affairs/Department of Defense) [6], ‘Treatment for Post-Traumatic Stress Disorder, Operational Stress Injury, or Critical Incident Stress: Clinical Practice Guidelines’ (Canadian Agency for Drugs and Technologies in Health CADTH) [7], ‘Post-traumatic stress disorder NICE guideline’ (National Institute for Health and Care Excellence NICE) [8] endorsed by the Royal College of Psychiatrists, and Phoenix Australia’s ‘Australian Guidelines for the Prevention and Treatment of Acute Stress Disorder, Posttraumatic Stress Disorder and Complex PTSD’ [9] endorsed by the Royal Australian and New Zealand College of Psychiatrists. There is consensus across these guidelines that trauma-focused psychological therapies of various modalities are recommended as first line approaches in the management of trauma-related disorders, particularly cognitive behavioural therapy (CBT), stress management and eye movement desensitization and reprocessing (EMDR). Psychotropic medications including selective serotonin reuptake inhibitors and serotonin norepinephrine reuptake inhibitors (specifically venlafaxine) are recommended as a second line treatment [4–9]. The World Health Organisation mhGAP ‘Guidelines for the Management of Conditions Specifically Related to Stress’ states that for the management of PTSD symptoms the use of ‘culturally appropriate interventions that are not harmful’ may be considered [4]. Only the US Veterans Affairs/Department of Defense ‘VA/DoD Clinical Practice Guidelines: Management of Posttraumatic Stress Disorder and Acute Stress Disorder’ makes specific reference to complementary therapies (CTs). These guidelines were developed particularly for the care of military veterans, and concluded that whilst certain CTs have shown promising results there is insufficient evidence to categorically recommend either for or against a range of CTs including acupuncture, creative arts therapies (e.g., music, art, dance), meditation, yoga, Qigong and Tai chi [6].

### Trauma-related Disorders Amongst Refugees - Epidemiology

Many people of refugee background and those seeking asylum have endured experiences of torture and trauma. The combination of forced migration, exposure to traumatic incidents, and acculturative stress associated with resettlement in a new country significantly elevates the risk of mental health issues among refugees [1, 10]. Estimates of the prevalence of PTSD in adult refugees have ranged from 3 to 86% and those for Major depressive disorder (MDD) have ranged from 3 to 80%. This high variation in prevalence can be attributed to heterogeneity between studies of diverse populations and using different measures [1]. A systematic review of 20 surveys involving 6,743 adult refugees from seven countries found a 9% prevalence of PTSD, which is roughly ten times higher than the age-matched general populations in those same countries [11]. Despite the significantly elevated prevalence, refugees often face limited and inadequate access to support systems [12]. Individuals seeking asylum are at heightened risk, particularly in the context of involuntary detention. In a 2016 study conducted across 25 detention centres operated by the Australian Government Department of Home Affairs (excluding Papua New Guinea and Nauru), it was demonstrated that roughly half of the detained refugee population reported symptoms indicative of PTSD. Additionally, one-third of children, adolescents and adults exhibited symptoms requiring specialist evaluation [13].

### Barriers to Receiving Treatment Faced by Refugees

Refugees face various barriers in accessing treatment such as limited resources, language and cultural differences, stigma and lack of familiarity with Western health systems. Bradley et al. reported that over 30% of these patients do not benefit from standard treatments [14]. Refugees often present with unique challenges, including trauma-related disorders and physical conditions resulting from torture and trauma [1, 14]. They may prefer to seek treatment aligned with their cultural beliefs and health care preferences which may include Western models of care in addition to complementary therapies and traditional medicines [1, 15].

### Definition of Complementary Therapy and Integrative Medicine

As defined by the World Health Organization (WHO), traditional medicine refers to the ‘sum total of the knowledge, skill, and practices based on the theories, beliefs, and experiences indigenous to different cultures, whether explicable or not, used in the maintenance of health as well as in the prevention, diagnosis, improvement or treatment of physical and mental illness.’ Complementary medicine refers to ‘health care practices that are not part of that country’s own tradition or conventional medicine and are not fully integrated into the dominant health-care system’ and is used ‘interchangeably with traditional medicine in some countries.’ Herbal medicine refers to the use of ‘herbs, herbal materials, herbal preparations and finished herbal products, that contain as active ingredients parts of plants, or other plant materials, or combinations’ [16]. Across the world there is a continuing increase in the use of herbal and plant-based medicines, with an estimated 80% of the world population utilizing them as part of primary health care treatment for a range of ailments [17].

Integrative medicine, which combines conventional medicine and CTs, is gaining recognition within the discipline of psychological medicine [18]. Various treatments have demonstrated effectiveness in managing mental health conditions including biological treatments, herbal remedies, psychological therapies, and mind-body approaches that are now accepted by mainstream practitioners [19–21]. There is also evidence that supports the adaptation of CBT for the management of anxiety and depressive disorders when working with migrants and refugees through the use of culturally accepted explanatory models or idioms of distress and the use of culturally appropriate techniques such as imagery, mindfulness, yoga and meditation, which may be particularly helpful in assisting patients to regulate emotion and address somatic symptoms of distress [22–24].

### Application of Complementary Therapies in Psychiatry

The use of CTs in the management of mental health conditions is gaining popularity across the world [16]. In the USA, a national household survey showed that herbal/natural medicines were the most utilised CTs for psychiatric disorders, with St John’s wort (Hypericum perforatum) and Kava (Piper methysticum) as a treatment of mood disorders being the most used in this population [25].

In 2021, Sarris et al. published a systematic review of the efficacy and effectiveness of plant-based medicines (phytoceuticals) in the treatment of psychiatric disorders. In this study, supportive evidence was found for St John’s wort for MDD, curcumin and saffron for MDD or depressive symptoms, and ginkgo for total and negative symptoms in schizophrenia. Their study identified several limitations in methodology as well as potential publication bias [25].

There is also growing evidence supporting the use of nutrient supplements (nutraceuticals) as adjunctive treatments in the management of a range of specific mental disorders, that is gaining acceptance in mainstream treatment and is beyond the scope of this literature review [26].

Mind-body interventions have also gained recognition in addressing trauma-related mental health issues, as they focus on bodily experiences and self-regulation [27]. Mindfulness has gained widespread acceptance and is now incorporated in various psychological therapies in Western practice. Yoga is increasingly used as a CT for psychiatric disorders including PTSD to help build self-regulation skills and bridge the mind-body disconnect [28, 29].

Despite the emerging evidence, combining CTs with Western treatments may have potential risks, especially if patients do not disclose all treatments. An Australian study by Byard et al. highlights the potential risks of combining certain herbal medicines, especially when combined with prescription medications. This includes the risk of serotonin syndrome, hepatic and renal failure, and exacerbation of pre-existing conditions [30]. Hence there is a need to understand the evidence for CTs in managing trauma-related disorders as well as the potential risks associated with combining CTs and pharmaceuticals, which can inform an integrative approach to care.

### The Place of Complementary Therapies in Refugee Mental Health Care

Many refugees are familiar with traditional remedies in their countries of origin, and they often search for traditional healers in their new country [31]. Herbal medicine, massage, and dietary therapies have been applied in various cultures worldwide [32].

It is widely agreed that the complexity of refugee health issues in Western countries necessitates diverse and flexible strategies [10, 33, 34]. Research supports medically pluralistic approaches that include both Western and traditional medicine in providing holistic health care for refugees [34, 35].

MacDuff et al. conducted a systematic review of the use of CT by refugees for all health conditions. A range of therapies were used including mind-body medicine, herbal remedies, manipulative therapies, and energy medicine. Most studies focused on Asian refugee populations (66%) and whilst mental health was not the specific focus of the review, mental problems related to trauma accounted for 36% of CT use [36].

A literature review by Longacre et al. in 2012 examined reports in the Western medical literature of various CT modalities in the care of refugees with trauma-related disorders [37]. Based on the results of a PubMed search of articles published until November 2011 there were three studies of the use of meditation, one of dance/movement, three of spiritually based healing, three of music therapy three of traditional Chinese medicine and acupuncture, one of Tai chi, one of Reiki, five of the integrated use of CT and three studies regarding the use of mind-body interventions. It was noted by the authors that ‘integrative medicine clinics may…provide effective forums for social and community development, and a novel space in which to offer other necessary resources, including language and social services.’ Hence integrative mental health services provide an opportunity for a culturally acceptable model of care in which patients may receive holistic treatment. The authors concluded that the efficacy of specific CT modalities in the treatment of survivors of torture and refugee trauma was largely unknown and that further research was warranted to assess the impact of particular CT modalities [37]. Hence there is a need for further research to document the current evidence base for integrative therapies in the management of trauma-related disorders specifically in the refugee population.

## Aim

This narrative review aims to inform clinical practice by exploring the evidence base for CTs in the management of trauma-related disorders amongst refugees by summarising the available research on the topic as described in the medical literature. It also aims to summarise the evidence base for integrative approaches of relevance to the management of trauma-related disorders in this population.

## Methods

The authors considered various types of literature reviews including a systematic review or a scoping review. The authors did not have a particular hypothesis prior to commencing the research and were particularly focused on reviewing the available medical literature in this field. Hence, a narrative review was chosen in order to collate an initial summary of the available peer-reviewed medical literature on CTs in the management of trauma-related disorders amongst refugees. A narrative review type was also selected to allow for the synthesis of a range of perspectives including studies with varying methodologies. The review followed certain PRISMA guidelines but only in the context of a narrative review as outlined [38].

### Eligibility Criteria

The study included peer-reviewed articles published in the English language using the date limits from earliest available publication to October 2023. Articles primarily focused on interventions provided by practitioners without reference to CTs and articles not specifically related to the care of refugees or displaced persons were excluded. Studies that were considered but ultimately excluded included those focusing on the role of certain therapies that are now integrated into mainstream medicine approaches including mindfulness based strategies and nutritional medicine. This was done on the basis that such practices are now accepted within mainstream medicine and hence were considered outside the scope of this narrative review.

### Information Sources

Given the authors’ particular focus on evaluating research published in the medical literature, the database Medline (via PubMed) was utilised to identify studies. A snowball search method was utilised by the primary author reviewing the bibliography at the end of relevant articles to identify other articles.

### Search Strategy

Suitable articles were searched based on the following MeSH terms: post-traumatic stress disorder AND refugee AND complementary therapies. Replacing the term post-traumatic stress disorder with the term trauma-related disorders did not find additional articles. Additional searches were conducted for post-traumatic stress disorder AND refugee AND specific forms of CTs including herbal medicine, yoga and acupuncture.

### Selection Process

49 articles were identified in the preliminary search. Using the snowball search method 33 additional articles were included. One article was removed before screening as it was retracted. 80 studies were retrieved and were assessed for eligibility. The process used to analyse the selected articles involved the first author reading each article and noting the type of complementary therapy used, the context in which it was used and the conclusions regarding the potential benefits of each type of CT.

**Fig. 1:**
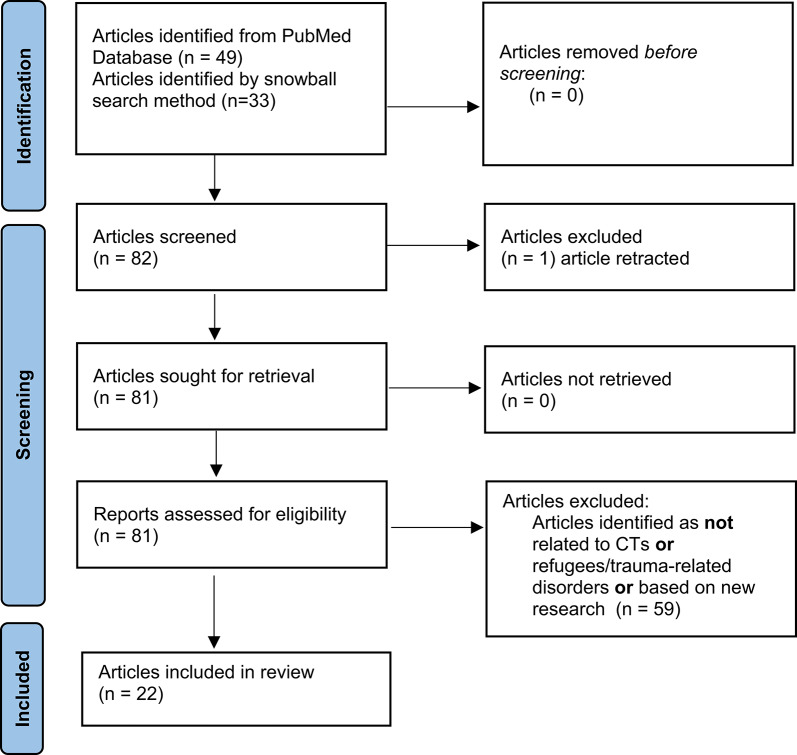
Flowchart outlining the search and selection strategy

After applying exclusion criteria for the narrative review and including only articles representing novel research, ultimately 22 articles were considered eligible for inclusion in the review as depicted in Fig. 1 (adapted from the PRISMA 2020 flow diagram) **(**Fig. 1**)** [38].

### Data Collection Process

The first author collected data from relevant articles by independently reviewing each article and summarising the following: type of study, study location by country, key findings including type of CT under investigation, number of participants and outcomes noted. Articles were grouped according to the modality of CT studied. These findings were independently reviewed by the second author and revised further in collaboration with the other co-authors.

Statistical analysis of effect measures was not undertaken and a formal bias assessment was not conducted.

## Results

The articles were categorised based on the modality of the CT. Results were at times challenging to compare due to methodological differences, varying time periods of studies and statistical reporting. A meta-analysis was not possible due to variability in quality of analysis and reporting, with many papers not reporting effect sizes or estimates of precision **(**Table 1**).**

**Table 1:** Summary of articles regarding the use of CTs in the management of trauma-related disorders amongst refugees

Title	First Author/Year	Country	Type of study/methods	Key Findings	Limitations	
*Herbal medicine*						
Complementary Medicine Use and Self-perceived Discrimination Among Asylum Seekers in Switzerland: A Cross-sectional Study	Walthert et al. 2020 [39]	Switzerland	Cross-sectional study	*n* = 61, asylum seekers (diverse backgrounds) 46% used CT; just under a third (28%) used CT within the previous year Herbal medicine was the most frequently used CT Only half of the respondents disclosed their CT use to their physician	Small sample size Selection bias (language barrier limited recruitment)	
*Yoga and Meditation*						
Calming the mind: Healing after mass atrocity in Cambodia	Agger 2015 [42]	Cambodia	Qualitative research	*n* = 27, Cambodian survivors of the Khmer Rouge regime Study of survivors of the Khmer Rouge regime in Cambodia noted the benefit of diverse and complex healing rituals derived from Khmer Buddhism Such practices extend beyond aspects of Eastern traditions such as meditation and mindfulness that are being incorporated into Western psychological therapy and are part of a richer cultural healing process	Data limited to individual interviews with the assistance of a translator	
Treating children traumatized by war and Tsunami: a comparison between exposure therapy and meditation-relaxation in North-East Sri Lanka	Catani et al. 2009 [45]	Sri Lanka	Randomized treatment comparison	*n* = 71, refugee children in North-East Sri Lanka UCLA PTSD Index (UPID) used to compare Narrative Exposure Therapy (NET) and Meditation-Relaxation treatments In both groups, PTSD symptoms and impairment in functioning significantly reduced at 1 month and remained stable at 6 months No significant difference between the two therapy groups at 1 month and 6 month follow-up PTSD symptom remission in 81% for the children in the NET group and 71% for those in the Meditation-Relaxation group at 6 months supports the beneficial effect of both interventions	Small sample No control group without any intervention No standard test for measurement of physical health and level of functioning	
Reduction in posttraumatic stress symptoms in Congolese refugees practicing transcendental meditation	Rees et al. 2013 [43]	USA	Pilot study	*n* = 21, Congolese refugees Posttraumatic Stress Disorder Checklist–Civilian (PCL-C) used to assess symptoms TM practice significantly reduced PTSD symptoms PCL-C scores decreased from an average of 65 at baseline to less than 30 on average after 30 days of TM practice and remained low after 135 days	PCL-C was the single test employed; the translated versions were not validated before the study Potential responder bias by offering food and personal care items to participants Compliance was not systematically assessed	
Significant reductions in posttraumatic stress symptoms in Congolese refugees within 10 days of Transcendental Meditation practice	Rees et al. 2014 [44]	USA	Pilot study	*n* = 11, Congolese refugees PCL-C used to assess symptoms Significant reduction in PCL-C score of on average 29.9 points from 77.9 to 48.0 in 10 days and a further reduction of an average of 12.7 points to 35.3 at 30 days TM practice may provide a prompt solution to address PTSD for those impacted by the trauma of war and violence	Single-group only design with a small number of subjects Absence of an active control group PCL-C is not validated in Lingala or Swahili Possible greater degree of help-seeking in participants (Congolese refugees living in temporary shelters in Uganda)	
Self-Practice of Stabilizing and Guided Imagery Techniques for Traumatized Refugees via Digital Audio Files: Qualitative Study	Zehetmair et al. 2020 [41]	Germany	Descriptive study	*n* = 42, adult asylum seekers (diverse backgrounds) Primary Care PTSD Screen for DSM-5 (PC-PTSD-5), two-item Patient Health Questionnaire (PHQ-2), short version of the General Anxiety Disorder questionnaire (GAD-2), Self-Assessment Manikin scale (SAM) and the distress thermometer of the Refugee Health Screener-15 (RHS-15 distress thermometer) were used to assess mental distress Audio-based stabilizing and guided imagery techniques were able to alleviate symptoms associated with mental distress in patients who continued to use self-practiced guided imagery techniques (about half of the cohort)	Possibility of a tendency towards compliant or socially desirable behaviour or answers High dropout rate (50%) Heterogeneous sample Selection bias The impact of medications or other stabilizing effects cannot be ruled out Self-perception of short term symptom relief assessed (but not measured on follow-up)	
*Art therapy*						
Beyond where it started: a look at the “Healing Images” experience	Goodsmith 2007 [47]	USA	Descriptive study	*n* = 30, refugees (diverse backgrounds) Subjective report of perceived benefits deriving from the “Healing Images” programme Participants reported benefit from the group as an escape from psychological and physical isolation and as a process of personal reflection	No comparison group No data available to demonstrate effectiveness	
A controlled early group intervention study for unaccompanied minors: Can Expressive Arts alleviate symptoms of trauma and enhance life satisfaction?	Meyer et al. 2017 [48]	Norway	Clinical trial	*n* = 145, asylum seeker children (diverse backgrounds) Manualised 5 session intervention incorporating principles of trauma intervention including promoting safety, calming, self-efficacy, connectedness and hope Hopkins Symptom Checklist-25 (HSCL-25) used as a measure of psychological distress and Harvard Trauma Questionnaire (HTQ) used as a measure of post-traumatic stress symptoms score Improvement in mental health complaints and life satisfaction and hope noted (although not statistically significant) A sense of group belonging and hope was noted amongst group members Group interventions with expressive arts may be a cost-effective supplement to basic care for unaccompanied minor refugees after arrival to a host country	Study participants not formally diagnosed with PTSD Only children participated Gender specific Allocation not completely randomised Extended follow-up period of 25 months although dropout noted The origin of refugees was mainly from two countries (Afghanistan and Somalia) that may limit the generalizability of the findings	
Trauma-Focused Art Therapy in the Treatment of Posttraumatic Stress Disorder: A Pilot Study	Schouten et al. 2019 [49]	Netherlands	Pilot study	*n* = 12, refugees (diverse backgrounds) PTSD symptom severity assessed using HTQ Mixed results including reduction in PTSD symptoms across all measures in 2 participants, decrease in avoidance amongst 5 participants whilst 4 participants demonstrated an increase in avoidance 25% dropout rate	12 participants including 10 refugees from diverse backgrounds and 2 other participants with a history of early childhood trauma (that may limit generalisability) Small sample size Absence of control condition PTSD symptoms assessed immediately after treatment without additional follow-up measurement Participants with comorbid depression excluded	
Group crisis intervention for children during ongoing war conflict	Thabet et al. 2005 [46]	Palestine	Non-randomised study	*n* = 111, Palestinian children Child Post-Traumatic Stress Disorder Reaction Index (CPTSD-RI) and Childhood Depression Inventory (CDI) used to assess PTSD and depressive symptoms No significant difference was noted between the 47 group intervention participants, 22 education about symptoms group participants and 42 control group participants Exposure to trauma and the non-active nature of the intervention possibly impacting the results	Not randomised Absence of diagnostic interviews Process of the intervention was not investigated The large size and developmental heterogeneity of the intervention groups may have impacted on the results PTSD and depression may have different treatment requirements Exposure to violence during the intervention period was not measured	
*Music therapy*						
Music therapy was noninferior to verbal standard treatment of traumatized refugees in mental health care: Results from a randomized clinical trial	Beck et al. 2021 [52]	Denmark	Randomised controlled trial	*n* = 74, refugees (diverse backgrounds) Trauma symptoms assessed using HTQ, quality of life assessed using the WHO Well-being Index (WHO-5), dissociative symptoms assessed using the Somatoform Dissociation Questionnaire (SDQ-20) and the Dissociative Symptoms Scale (DSS), and adult attachment assessed using the Revised Adult Attachment Scale (RAAS) Music therapy proved to be noninferior to the treatment as usual group (provided with standard psychological therapy) Much higher retention rates were observed in the music therapy group than in the active control (5% drop out rate compared with 40%) Treatment effect was seen immediately after treatment and at 6 month follow-up	Concurrent use of pharmacological treatments may have impacted on findings Intervention did not follow a fixed protocol with established validity Unblinded collection and statistical analysis of secondary outcomes and the absence of a systematic assessment of self-rated treatment fidelity	
A pilot analysis of the psychological themes found during the CARING at Columbia–Music Therapy program with refugee adolescents from North Korea	Choi 2010 [54]	South Korea	Qualitative research	*n* = 9, North Korean refugees 30% diagnosed with PTSD Participants were able to express their emotions through arts medium Themes emerged during the program: avoidance, distrust, loneliness, loss, and fear	Data obtained from field notes and anecdotal reports written by music therapists Small sample size	
Comparing the effect of social media-based drama, music and art therapies on reduction in post-traumatic symptoms among Nigerian refugees of Russia’s invasion of Ukraine	Gever 2023 [50]	Nigeria	Non-randomised study	*n* = 313, Nigerian refugees International Trauma Questionnaire (ITQ) used to measure PTSD symptoms Statistically significant reduction in ITQ score in the intervention group to a mean of 14.8 compared with the control group with a mean of 28.2 Social media-based therapies effectively reduced PTSD symptoms Drama therapy was more effective than art and music therapies	Lack of a follow-up assessment Did not determine if gender played a role in the effectiveness of the intervention Potential generalisability of findings limited	
The Effect of Relaxation Music Listening on Sleep Quality in Traumatized Refugees: A Pilot Study	Jespersen et al. 2012 [53]	Denmark	Pilot study	*n* = 15, refugees (diverse backgrounds) Sleep quality assessed using the Pittsburgh Sleep Quality Index (PSQI), trauma symptoms assessed using the PTSD-8 questionnaire and well-being assessed using the “How Do You Feel?” questionnaire (HDYF) Significant change in global sleep quality from pre to post intervention was demonstrated in the music therapy group despite the fact that there was no improvement in trauma symptoms in either group Results support the use of relaxation music listening at bedtime to improve sleep quality in traumatized refugees	High dropout rate (21%) Small sample size Uneven number of participants in the music therapy and control group Short time span Blinding not possible Possibility of treatment bias	
*Dance/movement therapy*						
Moving Through the Trauma: Dance/Movement Therapy as a Somatic-Based Intervention for Addressing Trauma and Stress Among Syrian Refugee Children	Grasser et al. 2019 [55]	USA	Pilot study	*n* = 25, Syrian refugees (children) University of California, Los Angeles Posttraumatic Stress Disorder Reaction Index (UCLA PTSD RI) used to assess mental distress Statistically significant decrease in self-reported mental health symptoms DMT may be a feasible way to help reduce stress and trauma-related symptoms in a community-based setting	Small sample size No control group, single group design, Potential improvement related to some mothers of children participating in yoga therapy	
Dance/movement therapy approaches to fostering resilience and recovery among African adolescent torture survivors	Harris 2007 [56]	USA and Sierra Leone	Descriptive study	2 groups, refugees (Sierra Leone and South Sudan) Two dance/movement therapy initiatives explored in groups of adolescents from Sierra Leone and South Sudan Both groups addressed management of anger and restoring interpersonal connection through traditional dancing and drumming Anecdotal support of reduction in average symptom expression	No control group Informal sessions with participants being youths from Sierra Leone and South Sudan (which may limit generalisability) No data available to demonstrate effectiveness	
*Qigong and Tai chi*						
Treating survivors of torture and refugee trauma: a preliminary case series using qigong and tai chi	Grodin 2008 [57]	USA	Case series	*n* = 4, refugees (diverse backgrounds) Preliminary observations from four cases supporting the potential efficacy of incorporating qigong and Tai chi into the treatment of survivors of torture and refugee trauma Improvement noted in psychological, cognitive, pain, sleep and physiological symptoms	Small sample size Informal methodology No control group All patients received concurrent psychiatric treatment and psychotherapy	
*Acupuncture*						
Supporting Communities in Humanitarian Crises with Acupuncture and Integrative Medicine A Perspective	Budd 2023 [60]	Greece	Case series	*n* = 3, refugees from Syria and Afghanistan Acupuncture was of benefit to three patients seeking asylum who arrived in Lesvos	Improvement in anxiety and PTSD symptoms based on subjective assessment by the clinician	
Acupuncture and traditional Chinese medicine for survivors of torture and refugee trauma: a descriptive report	Highfield et al. 2012 [58]	USA	Descriptive study	*n* = 50, refugees (diverse backgrounds) Patients accepted the treatments with no refusals and continued to request to return for follow up treatments for months Pain assessed using a 10-point numeral analogue scale 56% reported a decrease in pain over the course of treatment; patients reported improvement in sleep and energy although these were not quantified Acupuncture may be a reasonable, feasible and positive method for addressing chronic pain and psychological distress of refugees and trauma survivors	Lack of complete medical histories from patients Lack of a cross-culturally relevant instrument to assess pain Lack of control group Focus on pain, and the ‘reward’ of more attention through more acupuncture potentially contributing to reporting higher pain levels Possibility of responder bias with participants reporting improvement to please providers Results limited by lack of complete patient records	
Acupuncture for refugees with posttraumatic stress disorder: initial experiences establishing a community clinic	Pease et al. 2009 [59]	USA	Case series	*n* = 16, refugees (diverse backgrounds) Acupuncture was well-received among the refugee population Reduction in PTSD symptoms in 14 of the 16 patients was noted based on clinical observations	Treatment was not standardized No control group Limited clinical observation	
*Neurofeedback*						
Neurofeedback as an adjunct therapy for treatment of chronic posttraumatic stress disorder related to refugee trauma and torture experiences: two case studies	Askovic et al. 2017 [61]	Australia	Case series	*n* = 2, refugees (unstated origin) PTSD symptoms measured using the HTQ, depression assessed using the Hopkins Symptom Checklist-Depression (HSCL-D), and anxiety assessed with the Hopkins Symptom Checklist-Anxiety (HSCL-A) Significant reduction in PTSD symptoms and improvement in daily functioning noted in 2 cases (one received 26 sessions and the other 36 sessions) Quantitative EEG measures indicated a normalisation of EEG markers relating to trauma, including overarousal at rest and working memory function	Small sample size Neurofeedback given in conjunction with trauma-informed psychiatric treatment and psychological therapy which may have impacted on interpretations of findings	
Evaluation of Neurofeedback for Posttraumatic Stress Disorder Related to Refugee Experiences Using Self-Report and Cognitive ERP Measures	Askovic et al. 2020 [62]	Australia	Pilot study	*n* = 13, refugees (diverse backgrounds) PTSD symptoms measured using the HTQ, symptoms of anxiety and depression assessed using the Hopkins Symptom Checklist-25 (HSCL-25) and cognitive control assessed using a visual continuous performance task (VCPT) Greater reduction of symptoms of PTSD in the neurofeedback group; 12 of the neurofeedback group had symptom reduction to below diagnostic threshold compared with 1 of the trauma counselling group	Small sample size Non-random allocation	

### Herbal Medicine

Herbal medicine refers to the use of medicinal plant products, and is a wide ranging discipline [19]. Despite the growing evidence base for the use of herbal medicine in psychiatry, only one study was identified using the aforementioned search terms. A cross-sectional study investigated the prevalence of the use of various forms of CT among asylum seekers in Switzerland and found that 46% of participants had used CT at some point in their lives, with herbal medicine being the most frequently employed form. Among the whole sample, 38% believed that herbal medicine had no risk or almost no risk of adverse events [39].

### Yoga and Meditation

Mindfulness practices and psychological therapies that incorporate mindfulness such as Acceptance and Commitment Therapy have gained mainstream acceptance [6]. Yoga and meditation practices have also gained prominence in Western countries as techniques that provide an adjunct to psychological therapy in addressing a variety of conditions. It has been suggested that such practices may have a role when working with resettled refugees, including movement-based and body-focused interventions as adjunctive therapeutic approaches to traditional Western treatments [40]. Five studies explored the application of yoga and meditation in the treatment of refugees. A descriptive study by Zehetmair et al. using qualitative interviews assessed the impact of self-practice stabilizing and guided imagery techniques. Participants were newly arrived adult asylum seekers with a diagnosis of PTSD. They were provided with audio files containing mindful breathing, body scan and guided imagery techniques. According to follow-up interviews conducted after nine days and two months, the techniques assisted patients in various aspects, including arousal, concentration, sleep, mood, thoughts, empowerment and tension. The study’s limitations included reliance on self-reports, selection bias and high dropout rate [41]. Agger’s qualitative study examined the ways in which Cambodians impacted by the Khmer Rouge regime used meditation in their recovery. 27 survivors of torture from the Khmer Rouge regime were interviewed, analysis of which suggested that complex Buddhist rituals and techniques of meditation may enhance feelings of security and well-being, thereby helping survivors cope with residual distress [42]. In a pilot study by Rees et al. the impact of Transcendental Meditation (TM) practice on Posttraumatic Stress Symptoms among Congolese refugees was examined using the Posttraumatic Stress Disorder Checklist–Civilian (PCL-C). All individuals in the TM group demonstrated clinically significant improvements in their PCL-C scores, while none of the participants in the control group exhibited such progress. The study acknowledged several limitations, including selection bias, the validity of the PCL-C test in participants’ languages and the lack of assessment of adherence to TM [43]. One year later, the same authors recruited part of the control group for the aforementioned study to assess whether TM practice would reduce PTS. Average PCL-C scores dropped from 77.9 to 48.0 in 10 days, then dropped another 12.7 points to 35.3 at 30 days. Effect size at 10 days was high (d = 4.05) [44]. Catani et al. randomly assigned children with diagnosis of PTSD in a refugee camp in North-East Sri Lanka (*n* = 71) to either Narrative Exposure Therapy for children (KIDNET) or meditation-relaxation (MED-RELAX). Outcome measures included severity of PTSD symptoms, level of functioning and physical health. In both treatment conditions, PTSD symptoms and impairment in functioning were significantly reduced at one-month follow-up and remained stable over time. There were no significant differences between the two therapy groups in any outcome measures [45].

### Art Therapy

Art therapy allows the individual to explore their emotions, develop self-awareness and improve emotional wellbeing through the creative process. There were five studies on art therapy amongst refugees which revealed mixed results. Thabet et al. studied the application of art therapy in children aged 9 to 15 years with symptoms of PTSD in the Gaza Strip. The participants were divided into three groups. The first group were encouraged to express their experiences and emotions through creative activities. The second group received education regarding PTSD symptoms. The third group did not receive any interventions. The Child Post-Traumatic Stress Reaction Index (PTSD RI) and the Children’s Depression Inventory tests were administered to all participants before and after the study. The results indicated no significant differences among the three groups. Possible explanations included the persistent exposure to trauma and the relatively passive nature of the intervention [46]. Goodsmith’s narrative study explored art therapy for survivors of trauma and torture refugees who were engaged in the “Healing Images” group program in the United States. Participants captured photos representing sources of comfort and created self-portraits reflecting their lives that were shared and discussed in the group. The program facilitated self-exploration and nurtured a supportive group environment with participants reporting subjective improvement [47]. In Norway, a five-week expressive arts program (EXIT) by Meyer et al. was compared with life as usual (LAU) in an arrival centre for unaccompanied refugee boys aged 15 to 18. The participants were followed up over 25 months. Whilst there was no significant difference between the groups with regards to change in symptoms, the study showed some differences in mental health complaints, life satisfaction and expectations throughout the period of follow-up with more positive outcomes particularly regarding life satisfaction and hope amongst the EXIT group. The study was limited by gender, non-random allocation, high dropout rates, and the impact of asylum application outcomes on the variables over time [48]. In a pilot study conducted by Schouten et al. involving 12 patients with PTSD of whom ten were refugees, trauma-focused art therapy yielded mixed results. The intervention involved three phases (stabilization and symptom reduction, trauma-focused exposure, as well as integration and meaning-making). Some participants of refugee background experienced a decrease in PTSD symptom severity as assessed using the Harvard Trauma Questionnaire (HTQ). An increase in symptom severity was noted amongst participants with a long-term history of traumatization, multiple traumatic experiences, comorbidity with severe depression, personality disorder and exposure to recent stressors. The study was limited by a small sample size and absence of a control condition [49]. More recently, the effectiveness of social media-based music, art and drama therapy in treating PTSD symptoms was investigated by Gever et al. In this study, Nigerian evacuees from the Russia-Ukraine war were randomly assigned to treatment and control groups. The study showed a significant effect of the treatment condition on reduction in PTSD symptoms as assessed using the International Trauma Questionnaire (ITQ) after controlling for pre-intervention scores F(1,327) = 147.826, *p* = 0.01. Comparatively, drama therapy was more effective than art and music therapies. The study’s limitations included the absence of a follow-up assessment and the failure to determine the impact of gender [50].

### Music Therapy

Music therapy is another therapeutic approach that utilises the creative process to help individuals deal with emotional distress and address their physical, emotional, cognitive and social needs. Four studies were identified regarding the use of music therapy in refugees. Following an elaboration of a research study design by Beck et al. in 2018 an article by the same authors in 2021 reported on a randomised clinical trial comparing music therapy to standard psychotherapy (TAU) for refugees in Denmark diagnosed with PTSD [51, 52]. Music therapy was found to be non-inferior to standard treatment in terms of its ability to decrease symptoms as measured by the HTQ. There were higher remission rates in the music therapy group than in the active control (remission of ten patients in the music group, and two in the TAU group (*p* = 0.185, Fisher’s exact test). Limitations included the concurrent use of pharmacological treatments and the absence of a protocol with established validity for intervention [51, 52]. Jespersen and Vuust explored the impact of relaxation music on sleep quality, experience of trauma symptoms and wellbeing. Fifteen refugees with trauma symptoms and sleep problems participated and were randomized into two groups. The intervention group received relaxing music played at night through a music player nestled in an ergonomic pillow, while the control group received the ergonomic pillow alone. The Pittsburgh Sleep Quality Index (PSQI), PTSD-8 questionnaire, and the ‘How Do You Feel?’ questionnaire (HDYF) were utilised. The results indicated an improvement in sleep quality and wellbeing within the intervention group, although there was no significant change in the PTSD-8 measure for either of the groups. The authors suggested that the lack of change in trauma symptoms might be attributed to the high levels of trauma symptoms among the participants, with 12 out of the 15 likely meeting the criteria for a diagnosis of PTSD [53]. In Choi’s qualitative study of a music therapy program for North Korean refugees aged 18 to 24, five common psychological themes (avoidance, distrust, loneliness, feelings of loss, and fear) were expressed by the participants over the course of the therapy that was perceived to be of benefit [54].

### Dance/Movement Therapy

Dance/movement therapy (DMT) is the therapeutic use of dance and movement to enhance the physical, emotional, cognitive and social wellbeing of an individual. In a pilot study conducted by Grasser et al. the impact of a 12-week DMT program on Syrian refugee children aged 7 to 14 was explored. The self-report UCLA PTSD RI questionnaire was administered pre- and post-intervention. The authors found a statistically significant decrease in the severity of PTSD and anxiety symptoms. The study’s limitations included a small sample size, the absence of a control group and the potential influence of mothers who participated in classes alongside children [55]. A descriptive study by Harris explored the use of DMT as a means of promoting recovery among two groups of adolescent survivors of war from Sierra Leone and South Sudan. Program evaluation revealed a significant reduction in symptoms of anxiety, depression, intrusive recollection, arousal, and aggression. Also, the group members’ expressions of their experiences through culturally adapted role-play demonstrated the success of the therapy in overcoming stigma and facilitating meaningful community reintegration [56].

### Qigong and Tai Chi

Traditional Chinese practices that incorporate mind–body practices include Qigong and Tai chi. A case series study by Gordin et al. reported four cases and a review of the literature using Qigong and Tai chi in refugee survivors of torture and trauma. Preliminary observations supported the potential efficacy of incorporating Qigong and Tai chi into the treatment of these patients who were refugees from diverse backgrounds including Tibet, Lebanon, Belarus and the Democratic Republic of Congo. All patients described improvement in physical pain, psychological distress and sleep. The study was limited by small sample size, informal methodology and the absence of a control group [57].

### Acupuncture

Acupuncture is a component of traditional Chinese medicine that involves the insertion and manipulation of needles into specific points on the skin, and has gained acceptance in many Western countries in the management of various physical and mental health conditions. Three studies investigated the impact of acupuncture on PTSD symptoms amongst refugees. Highfield et al. performed a retrospective study on the use of acupuncture on traumatised refugees. Out of 50 refugees at a major tertiary teaching hospital, 40% carried the diagnosis of PTSD. The primary complaint of all patients was chronic pain (100%). Using the Wong-Baker Faces Pain scale, 56% of patients reported a decrease in pain over the course of acupuncture. The use of acupuncture appeared to be acceptable to survivors and refugees from different backgrounds with PTSD. The study had multiple limitations including incomplete medical histories, absence of a control group and the possible impact of secondary gain [58]. In a case report series by Pease et al. 16 refugees with a diagnosis of PTSD received acupuncture. Chronic pain was the most common associated complaint. Authors reported considerable reduction in PTSD symptoms in 14 of the 16 patients based on clinical observations [59]. Budd reported on the experience of providing acupuncture treatment to three refugees who were victims of torture and trauma and who arrived by boat on the Greek island of Lesvos. The patients presented with a range of physical complaints including digestive problems, back pain, as well as anxiety, insomnia and PTSD symptoms. Based on clinical assessment it was noted that patients experienced some relief from acupuncture although no further data including objective assessments were obtained [60].

### Neurofeedback

Neurofeedback is a model of biofeedback that utilises EEG monitoring to promote more efficient brain functioning. Two Australian studies by Askovic et al. investigated the use of neurofeedback in refugees with PTSD. Preliminary case reports in 2017 described the application of neurofeedback in two refugees with chronic PTSD. Symptoms of PTSD, anxiety, and depression were measured by HTQ, HSCL-D and HSCL-A questionnaires. Both patients achieved clinically significant reductions in their symptoms of PTSD and improvement in daily functioning. Additionally, quantitative EEG measures indicated a normalisation of EEG markers related to trauma, including overarousal and working memory [61]. In the second study conducted in 2020, the use of neurofeedback combined with trauma counselling (NFT) was compared with trauma counselling alone (TC) in 13 adult refugees with chronic PTSD. NFT participants showed significantly lower symptoms as measured using the HTQ compared with the TC group, with a reduction of PTSD symptoms to below the diagnostic threshold in 12 of 13 participants. The authors postulated the impact of remediation of cognitive control processes [62].

## Discussion

### Summary of the Main Findings

Despite variation in the methods and quality of the data available, this review highlights that a broad range of CTs have demonstrated some evidence in the management of trauma-related disorders amongst refugees. A range of studies investigated the following modalities in the care of refugees: yoga and meditation (*n* = 5), music therapy (*n* = 4), art therapy (*n* = 4), acupuncture (*n* = 3), dance/movement therapy (*n* = 2), neurofeedback (*n* = 2), herbal medicine (*n* = 1) as well as Qigong and Tai chi (*n* = 1).

#### Review of Methods and Analysis

In the studies reviewed, various methods of research were employed, including pilot study (*n* = 6), descriptive study (*n* = 5), case report/series (*n* = 4), RCT/randomised treatment comparison (*n* = 2), non-randomised study (*n* = 2), qualitative research (*n* = 2) and clinical trial (*n* = 1). The heterogeneity of the studies with regards to type, methodology, design and demographics limits comparison. However, all but one study suggested that interventions improved participants’ distress in some way.

### Implications for Practice

This narrative review has certain implications for clinical practice. A significant number of refugees with trauma-related disorders remain impacted due to high levels of symptomatology [63]. It is possible that such severe symptoms are more resistant to change. It is also recognised that patients of refugee background may not complete the full course of conventional treatments [1]. Studies on the use of psychotherapy (e.g. EMDR) for traumatised asylum seekers and refugees have reported high dropout numbers [64]. The complex nature of refugee trauma suggests that adequate therapy is likely to require a specialised and multifaceted approach.

Increasingly in countries of settlement for refugees, practitioners are faced with the challenge of developing treatment strategies for chronic conditions that are culturally applicable to patients of diverse backgrounds [65]. This narrative review addresses certain questions as to whether specific CTs have universal applicability or if their use and acceptance by patients may be determined by their cultural beliefs and practices.

As noted, meditation and mindfulness principles have been incorporated into Western psychological therapies. Whilst this is no doubt positive and will benefit many patients including refugees, the use of yoga and other traditional approaches may be more nuanced. This is highlighted in the study by Agger which demonstrates that elaborate Buddhist rituals and techniques were perceived to be of value to Cambodians impacted by trauma during the Khmer Rouge regime that extends beyond the use of certain techniques framed within Western psychological therapies [42]. The study by Catani et al. also demonstrated that amongst refugee children in Sri Lanka, meditation-relaxation therapy was as effective as Narrative Exposure Therapy and led to reduction amongst the group in severity of PTSD symptoms as well as improvement in functioning [45]. This is of particular note given the emphasis placed on the use of evidence-based guidelines that favour the use of Western psychological therapies, even in diverse settings. It is important that practitioners remain open to the experiences of refugees themselves based on their attitudes and understanding of traditional practices.

Nonetheless, this narrative review supports the notion that many modalities of CTs do have general applicability and may be of benefit to refugees from diverse backgrounds. As noted in this narrative review, several modalities were highly accepted by refugee participants, indicating that their use is consistent with culturally responsive care.

In total ten studies included in this narrative review explored the use of dance/movement therapy, art therapy and music therapy with overall positive outcomes, suggesting that such approaches may be adapted to refugees from diverse backgrounds rather than being confined to a particular cultural group [46–56].

Certain modalities of CT may lend themselves particularly well to an integrative approach within healthcare settings in order to provide holistic care. The two studies by Rees et al. are significant in that they were able to demonstrate improvements in mental distress amongst Congolese refugees using yoga and TM, despite the fact that these practices are not derived from their own culture [43, 44]. The study by Grodin et al. is also of note for its investigation into the use of Qigong and Tai chi in a small case series. One of the patients was of Tibetan background, for whom Qigong and Tai chi may be anticipated to be a potentially acceptable mode of treatment. However this modality of CT was also perceived to benefit the other participants from cultures where Qigong and Tai chi are not typically practiced [57]. Similarly, three studies included in this review investigated the role of acupuncture amongst a total of 69 patients of diverse backgrounds [58, 59, 60]. Although these studies adopted different methodologies, this narrative review suggests that acupuncture was widely accepted by refugees from diverse backgrounds and its use was associated with improvement in physical symptoms of pain as well as improvement in trauma-related psychological distress.

The role of herbal medicine in the treatment of various psychiatric disorders has become more established over the past decade. As noted by Walthert et al. herbal medicine was used by just under half of the 61 asylum seekers interviewed in a study in Switzerland and were considered to be well tolerated [39]. Although this was the only study identified by this narrative review that addressed the use of herbal medicine specifically in the management of trauma-related disorders amongst refugees, it is likely that the growing evidence base regarding herbal medicine would have clinical relevance to this group. In a systematic review by Sarris et al., high-quality evidence was found for the use of Piper methysticum (Kava), Passiflora spp. (passionflower) and Galphimia glauca (galphimia) for anxiety disorders [66]. Additionally, Hypericum perforatum (St John’s wort) and Crocus sativus (saffron) showed efficacy for the management of MDD. Other herbal medicines with preliminary evidence include Curcuma longa (turmeric) in depression, Withania somnifera (ashwagandha) in affective disorders, and Ginkgo biloba (ginkgo) as an adjunctive treatment in schizophrenia [66]. The same authors subsequently conducted a meta- analysis that gave supportive evidence for St John’s wort for MDD, curcumin and saffron for MDD or depression symptoms, and ginkgo for total and negative symptoms in schizophrenia. The study identified several limitations in methodology and reporting as well as potential publication bias [25]. It is estimated that approximately half of all refugees use traditional medicines for the management of health conditions in general, and only half of them disclose such use to medical providers [67, 68]. Hence it is important that all clinicians ask about the use of herbal medicine, notably due to potential risks of interactions and safety [21].

There is therefore a clear need for integrative approaches in the care of refugees. The Victorian Foundation for Survivors of Torture (Foundation House) (VFST) is a leading torture and trauma mental health service for refugees and asylum seekers in Melbourne, Australia, that was established in 1987 and is an example of a refugee health service providing CTs as part of an integrated treatment plan for refugees with mental health conditions [69]. CTs have been incorporated as a key component of trauma-focused care to patients at VFST since 1989. The CT service contributes to patients’ recovery through the provision of a range of CTs by experienced practitioners including massage, Shiatsu, naturopathy, acupuncture, yoga and other body-based therapies. The CT practitioners work closely with the assistance of interpreters and collaborate with Counsellors who provide psychological therapies. In a 2011 study, the role of CTs in an integrated model at VFST explored refugee women’s experiences with CTs, as well as psychotherapists’ understandings of CTs and their referral practices. The findings supported the inclusion of culturally familiar health care practices in the form of CTs [69]. According to Singer and Adams, “these therapies give primacy to caring for the complexity that is the ‘refugee body’: the amalgamation of physical pain with the complex social, political, and cultural factors that define the refugee experience” [69]. It is likely that CTs are culturally acceptable for refugees for other reasons. As noted by Schouten et al., in patients with verbal expression impediments CTs that utilise non-verbal techniques may be more acceptable and better tolerated. Various forms of touch-based CTs may assist refugees, particularly those with somatic symptoms of distress who may not be ready to engage in psychological therapy. Provided that they are administered in a culturally responsive way such CTs provide a valuable adjunct to other therapies and can also help with overcoming stigma [49]. CTs are an important adjunct to biomedical treatment options and psychological therapy for refugees in the delivery of an integrative approach to healthcare [70]. Central to such an approach is the effective collaboration between practitioners with diverse areas of specialisation who are able to work together to assist refugees in a holistic manner.

Furthermore, the majority of studies included in this narrative review described research conducted amongst refugees in either transition or resettlement countries (Table 1). Two studies reported on the benefits of yoga and meditation in Cambodia [42] and Sri Lanka [45] and one study reported the benefit of dance/movement therapy in Sierra Leone [56]. The study by Thabet et al. is of note in that there was no demonstrated impact of art therapy on PTSD and depressive symptoms amongst Palestinian children in Gaza. The authors suggested that the ongoing exposure to conflict may have been a contributing factor [46]. Given the known limitations of conventional Western treatments in circumstances where individuals face continuing exposure to significant hardship, it would be worthwhile to consider the evidence regarding the role of CTs in such contexts.

### Recommendations

On the basis of this narrative review it is recommended that mental health professionals support access to CTs when treating refugees from diverse backgrounds. There is an opportunity for mental health professionals to be aware of CT modalities and provided there are no contraindications including interactions with herbal medicines, to encourage refugee patients to engage in therapies that may be culturally acceptable. Understanding CTs can also enhance a holistic approach by filling therapeutic gaps in existing practices, treating ‘the whole person’ and increasing healthcare choices.

Further research would be warranted to substantiate the integration of CTs in the management of trauma-related disorders amongst refugees. In particular, further research regarding the specific modalities of CT outlined in this narrative review as applied to the care of refugee patients is warranted. In addition, qualitative research to explore refugees’ attitudes, preferences and experiences would be worthwhile, particularly in order to determine the cultural applicability of the various specific modalities of CT.

Another key recommendation to emerge is the need for a systematic review to better inform clinical practice. This narrative review focused on CTs in general. It would be worthwhile to undertake a systematic review regarding the evidence base by expanding the search terms to include each distinct type of CT that emerged from this narrative review including herbal medicine, dance/movement therapy, art therapy, music therapy, yoga and meditation, Qigong and Tai chi, acupuncture and neurofeedback. Such research may help to understand whether or not treatment with individual modalities is supported by evidence. As an initial step, the authors recommend that further research be considered including a scoping review as a precursor to a systematic review in order to ensure that adequate numbers of relevant studies are available for inclusion.

## Limitations

A key limitation of this narrative review is that only articles sourced from Medline were included. A systematic review preceded by a scoping review may yield additional research of relevance to this topic. The authors recognise that the selection of articles in this narrative review is biased given that Medline was the only database accessed and that high quality research articles that may be identified in other databases including Scopus, Web of Science, SocINDEX and EBSCO used in social sciences, humanities and education as well as sources from the grey literature were not included. As noted, the primary purpose of this narrative review was to assess the peer reviewed medical literature in this field.

An additional limitation of this narrative review is that the search terms for this literature review included CTs in general, as well as certain specific modalities that are well known including herbal medicine, yoga and acupuncture. Although a snowball search method was utilised to identify other articles, it is acknowledged that research focusing on other modalities of CTs may have been missed in this process.

With regards to the research studies included in this review, methodological limitations were noted by many researchers which also impacted on the findings of the narrative review. In most cases authors acknowledged that the quality of the evidence was constrained by small sample sizes and the absence of a control group, with limited numbers of RCTs. Several factors may explain these limitations. Many refugees who have endured traumatic experiences such as persecution may be hesitant to trust authorities, including healthcare workers engaged in research. Additionally the complexity of trauma symptoms, acculturation challenges and the impact of interpreters on the therapeutic relationship must also be considered. Furthermore, it is foreseeable that other culturally responsive approaches to integrative medicine that relate to the care of people from culturally and linguistically diverse backgrounds are also relevant in the care of refugees [15]. However, as noted, the focus of this literature review specifically centred on the management of trauma-related disorders amongst refugees.

## Conclusion

This narrative review offers some evidence that supports the use of CTs in the treatment of trauma-related disorders including PTSD in refugees. Various modalities have been reported to be of benefit including herbal medicine, dance/movement therapy, art therapy, music therapy, yoga and meditation, Qigong and Tai chi, acupuncture and neurofeedback. Further research with larger studies and thorough methodologies is needed to build the evidence base regarding the integration of CTs and conventional treatments including psychological therapy and pharmacotherapy.

## References

[1] Kaplan I. Rebuilding shattered lives: integrated trauma recovery for people of refugee background (2nd ed). The Victorian Foundation for Survivors of Torture Inc.: Brunswick, Australia 2020.

[2] American Psychiatric Association. Diagnostic and statistical manual of mental disorders, Text revision (5th ed.TR). American Psychiatric Association Publishing: Arlington, USA 2022.

[3] World Health Organisation. International Classification of Diseases, Eleventh Revision (ICD-11), World Health Organization (WHO); 2019/2021 Available from: https://icd.who.int/browse11 [Last accessed: 12/10/23].

[4] World Health Organization. Guidelines for the management of conditions specifically related to stress. 2013. Available from: https://www.who.int/publications/i/item/9789241505406 [Last accessed: 12/10/23]. 24049868

[5] American Psychological Association. Clinical Practice Guideline for the Treatment of PTSD. 2017. Available from: https://www.apa.org/ptsd-guideline [Last accessed: 12/10/23].

[6] US Veterans Affairs / Department of Defense. VA/DoD Clinical Practice Guidelines: Management of Posttraumatic Stress Disorder and Acute Stress Disorder. 2023. Available from: https://www.healthquality.va.gov/guidelines/MH/ptsd [Last accessed: 12/10/23].

[7] Canadian Agency for Drugs and Technologies in Health. CADTH Treatment for Post-Traumatic Stress Disorder, Operational Stress Injury, or Critical Incident Stress: A Summary of Clinical Practice Guidelines. CADTH. 2023. Available from: https://www.cadth.ca/treatment-post-traumatic-stress-disorder-operational-stress-injury-or-critical-incident-stress [Last accessed: 12/10/23]. 26180857

[8] National Institute for Health and Care Excellence. Post-traumatic stress disorder NICE guideline. 2018. Available from: www.nice.org.uk/guidance/ng116 [Last accessed: 12/10/23].

[9] Phoenix Australia. Australian Guidelines for the Prevention and Treatment of Acute Stress Disorder, Posttraumatic Stress Disorder and Complex PTSD. Phoenix Australia. 2023. Available from: https://www.phoenixaustralia.org/wp-content/uploads/2022/11/PTSD-Guidelines-Chapter-6-Treatment-recommendations.pdf [Last accessed: 12/10/23].

[10] Hollifield M, Warner TD, Lian N, et al. Measuring trauma and health status in refugees: a critical review. JAMA. 2002. 10.1001/jama.288.5.611. 12150673 10.1001/jama.288.5.611

[11] Fazel M, Wheeler J, Danesh J. Prevalence of serious mental disorder in 7000 refugees resettled in Western countries: a systematic review. Lancet. 2005;365(9467):1309–14. 10.1016/S0140-6736(05)61027. 15823380 10.1016/S0140-6736(05)61027-6

[12] Royal Australian and New Zealand College of Psychiatrists. The Provision of Mental Health Services for Asylum Seekers and Refugees. 2017; Available from: https://www.ranzcp.org/clinical-guidelines-publications/clinical-guidelines-publications-library/the-provision-of-mental-health-services-for-asylum-seekers-and-refugees [Last accessed: 12/10/23].

[13] Young P, Gordon MS. Mental health screening in immigration detention: a fresh look at Australian government data. Australas Psychiatry. 2016;24(1):19–22. 10.1177/1039856215624247. 26755798 10.1177/1039856215624247

[14] Bradley R, Greene J, Russ E, et al. A multidimensional meta-analysis of psychotherapy for PTSD. Am J Psychiatry. 2005;162(2):214–27. 10.1176/appi.ajp.162.2.214. 15677582 10.1176/appi.ajp.162.2.214

[15] Andary L, Stolk Y, Klimidis S. Assessing mental health across cultures. Bowen Hills, Australia: Australian Academic Press Pty. Ltd.; 2003.

[16] World Health Organization. Traditional, complementary, and integrative medicine. 2023. Available from: https://www.who.int/health-topics/traditional-complementary-and-integrative-medicine#tab=tab_1 [Last accessed: 12/10/23].

[17] National Center for Complementary and Integrative Health. What does NCCIH do?. 2023. Available from: https://www.nccih.nih.gov [Last accessed: 12/10/23].

[18] Lake J, Helgason C, Sarris J. Integrative mental health (IMH): paradigm, research, and clinical practice. Explore. 2012;8(1):50–7. 10.1016/j.explore.2011.10.001. 22225934 10.1016/j.explore.2011.10.001

[19] Sarris J, Moylan S, Camfield DA, et al. Complementary medicine, exercise, meditation, diet, and lifestyle modification for anxiety disorders: a review of current evidence. Evidence-based complementary and alternative medicine. 2012; 809653. 10.1155/2012/809653. 22969831 10.1155/2012/809653 PMC3434451

[20] Sarris J, O’Neil A, Coulson CE, et al. Lifestyle medicine for depression. BMC Psychiatry. 2014;14: 107. 10.1186/1471-244X-14-107. 24721040 10.1186/1471-244X-14-107 PMC3998225

[21] Clinical use. Of nutraceuticals in the adjunctive treatment of depression in mood disorders. Australasian Psychiatry. 2017;25(4):369–72. 10.1177/1039856216689533. 28135835 10.1177/1039856216689533

[22] Hinton DE, Rivera EI, Hofmann SG, et al. Adapting CBT for traumatized refugees and ethnic minority patients: examples from culturally adapted CBT (CA-CBT). Transcult Psychiatry. 2012;49(2):340–65. 10.1177/1363461512441595. 22508639 10.1177/1363461512441595

[23] Hinton D, Patel A. Cultural adaptations of cognitive behavioral therapy. Psychiatr Clin North Am. 2017;40(4):701–14. 10.1016/j.psc.2017.08.006. 29080595 10.1016/j.psc.2017.08.006

[24] Kananian S, Soltani Y, Hinton D, Niv N, Shatkin JP, Hamilton AB et al. Culturally Adapted Cognitive Behavioral Therapy Plus Problem Management (CA-CBT+) With Afghan Refugees: A Randomized Controlled Pilot Study. Journal of Traumatic Stress. The use of herbal medications and dietary supplements by people with mental illness. Community Mental Health Journal 2010: 46(6): 563–569; 10.1007/s10597-009-9235-2.

[25] Sarris J, Marx W, Ashton MM, et al. Plant-based medicines (Phytoceuticals) in the treatment of psychiatric disorders: A Meta-review of Meta-analyses of randomized controlled trials. Can J Psychiatry. 2021;66(10):849–62. 10.1177/0706743720979917. 33596697 10.1177/0706743720979917 PMC8573706

[26] Firth J, Teasdale SB, Allott K, et al. The efficacy and safety of nutrient supplements in the treatment of mental disorders: a meta-review of meta-analyses of randomized controlled trials. World Psychiatry. 2019;18(3):308–24. 31496103 10.1002/wps.20672 PMC6732706

[27] Van der Kolk BA. The Body Keeps the Score: Brain, Mind, and Body in the Healing of Trauma. New York, USA: Viking; 2014.

[28] Metcalf O, Varker T, Forbes D, et al. Efficacy of fifteen emerging interventions for the treatment of posttraumatic stress disorder: a systematic review. J Trauma Stress. 2016;29(1):88–92. 10.1002/jts.22070. 26749196 10.1002/jts.22070

[29] Van der Kolk BA. Clinical implications of neuroscience research in PTSD. Ann N Y Acad Sci. 2006;1071:277–93. 10.1196/annals.1364.022. 16891578 10.1196/annals.1364.022

[30] Byard RW, Musgrave I, Maker G, et al. What risks do herbal products pose to the Australian community? Med J Aust. 2017;206(2):86–90. 10.5694/mja16.00614. 28152355 10.5694/mja16.00614

[31] Sargent C, Marcucci J. Aspects of Khmer medicine among refugees in urban America. Med Anthropol Q. 1984;16(1):7–9. 10.1111/j.1937-6219.1984.tb00948.x.

[32] McIntyre E, Saliba AJ, Moran CC. Herbal medicine use in adults who experience anxiety: a qualitative exploration. Int J Qual Stud Health Well-being. 2015;10: 29275. 10.3402/qhw.v10.29275. 26680418 10.3402/qhw.v10.29275 PMC4683991

[33] Grodin MA, Piwowarczyk L, Fulker D, et al. Treating survivors of torture and refugee trauma: a preliminary case series using qigong and t’ai Chi. J Altern Complement Med. 2018;14(7):801–6. 10.1089/acm.2007.0736. 10.1089/acm.2007.0736 PMC2745908 18803491

[34] Watters C. Emerging paradigms in the mental health care of refugees. Soc Sci Med. 2001;52(11):1709–18. 10.1016/s0277-9536(00)00284-7. 11327142 10.1016/s0277-9536(00)00284-7

[35] Benedict A, Mancini L, Grodin M. Struggling to meditate: contextualizing integrated treatment of traumatized Tibetan refugee monks. Mental Health Relig Cult. 2009;12:485–99. 10.1080/13674670902788908.

[36] MacDuff S, Grodin MA, Gardiner P. The use of complementary and alternative medicine among refugees: a systematic review. J Immigr Minor Health. 2011;13(3):585–99. 10.1007/s10903-010-9318-8. 20224938 10.1007/s10903-010-9318-8

[37] Longacre M, Silver-Highfield E, Lama P, et al. Complementary and alternative medicine in the treatment of refugees and survivors of torture: a review and proposal for action. Torture. 2012;22(1):38–57. 23086004

[38] Page MJ, McKenzie JE, Bossuyt PM, et al. The PRISMA 2020 statement: an updated guideline for reporting systematic reviews. BMJ. 2021;372:71. 10.1136/bmj.n71 PMC8005924 33782057

[39] Walthert L, Bodenmann P, Burnand B, et al. Complementary medicine use and self-perceived discrimination among asylum seekers in Switzerland: a cross-sectional study. J Immigr Minor Health. 2020;22(1):61–5. 10.1007/s10903-019-00895-5. 31079290 10.1007/s10903-019-00895-5

[40] Kumar GS, Soffer G, Begg D. Movement-based therapies for resettled refugee populations in the United States. Int J Yoga Ther. 2021;31(1):24. 10.17761/2021-D-20-00043. 10.17761/2021-D-20-00043 34260691

[41] Zehetmair C, Nagy E, Leetz C, et al. Self-practice of stabilizing and guided imagery techniques for traumatized refugees via digital audio files: qualitative study. J Med Internet Res. 2020;22(9): e17906. 10.2196/17906. 32965229 10.2196/17906 PMC7542415

[42] Agger I. Calming the mind: healing after mass atrocity in Cambodia. Transcult Psychiatry. 2015;52(4):543–60. 10.1177/1363461514568336. 25653141 10.1177/1363461514568336 PMC4532676

[43] Rees B, Travis F, Shapiro D, et al. Reduction in posttraumatic stress symptoms in Congolese refugees practicing transcendental meditation. J Trauma Stress. 2013;26(2):295–8. 10.1002/jts.21790. 23568415 10.1002/jts.21790

[44] Rees B, Travis F, Shapiro D, et al. Significant reductions in posttraumatic stress symptoms in Congolese refugees within 10 days of transcendental meditation practice. J Trauma Stress. 2014;27(1):112–5. 10.1002/jts.21883. 24515537 10.1002/jts.21883

[45] Catani C, Kohiladevy M, Ruf M, et al. Treating children traumatized by war and tsunami: a comparison between exposure therapy and meditation-relaxation in North-East Sri Lanka. BMC Psychiatry. 2009;9: 22. 10.1186/1471-244X-9-22. 19439099 10.1186/1471-244X-9-22 PMC2685130

[46] Thabet AA, Vostanis P, Karim K. Group crisis intervention for children during ongoing war conflict. Eur Child Adolesc Psychiatry. 2005;14(5):262–9. 10.1007/s00787-005-0466-7. 15981138 10.1007/s00787-005-0466-7

[47] Goodsmith L. Beyond where it started: a look at the healing images experience. Torture. 2007;17(3):222–32. 19289895

[48] Meyer DeMott MA, Jakobsen M, Wentzel-Larsen T, et al. A controlled early group intervention study for unaccompanied minors: can expressive arts alleviate symptoms of trauma and enhance life satisfaction? Scand J Psychol. 2017;58(6):510–8. 10.1111/sjop.12395. 29105124 10.1111/sjop.12395

[49] Schouten KA, van MATh S, Knipscheer JW, et al. Trauma-Focused Art therapy in the treatment of posttraumatic stress disorder: A pilot study. J Trauma Dissociation. 2019;20(1):114–30. 10.1080/15299732.2018.1502712. 30111254 10.1080/15299732.2018.1502712

[50] Gever VC, Iyendo TO, Obiugo-Muoh UO, et al. Comparing the effect of social media-based drama, music and Art therapies on reduction in post-traumatic symptoms among Nigerian refugees of russia’s invasion of Ukraine. J Pediatr Nurs. 2023;68:e96–102. 10.1016/j.pedn.2022.11.018. 36470757 10.1016/j.pedn.2022.11.018

[51] Beck BD, Lund ST, Søgaard U, et al. Music therapy versus treatment as usual for refugees diagnosed with posttraumatic stress disorder (PTSD): study protocol for a randomized controlled trial. Trials. 2018;19(1): 301. 10.1186/s13063-018-2662-z. 29848343 10.1186/s13063-018-2662-z PMC5977477

[52] Beck BD, Meyer SL, Simonsen E, et al. Music therapy was noninferior to verbal standard treatment of traumatized refugees in mental health care: results from a randomized clinical trial. Eur J Psychotraumatol. 2021;12(1): 1930960. 10.1080/20008198.2021.1930960. 34285768 10.1080/20008198.2021.1930960 PMC8266250

[53] Jespersen KV, Vuust P. The effect of relaxation music listening on sleep quality in traumatized refugees: a pilot study. J Music Ther. 2012;49(2):205–29. 10.1093/jmt/49.2.205. 26753218 10.1093/jmt/49.2.205

[54] Choi CM. A pilot analysis of the psychological themes found during the CARING at Columbia–music therapy program with refugee adolescents from North Korea. J Music Ther. 2010;47(4):380–407. 10.1093/jmt/47.4.380. 21488604 10.1093/jmt/47.4.380

[55] Grasser LR, Al-Saghir H, Wanna C, et al. Moving through the trauma: dance/movement therapy as a somatic-based intervention for addressing trauma and stress among Syrian refugee children. J Am Acad Child Adolesc Psychiatry. 2019;58(11):1124–6. 10.1016/j.jaac.2019.07.007. 31348987 10.1016/j.jaac.2019.07.007

[56] Harris DA. Dance/movement therapy approaches to fostering resilience and recovery among African adolescent torture survivors. Torture. 2007;17(2):134–55. 17728491

[57] Grodin MA, Piwowarczyk L, Fulker D, et al. Treating survivors of torture and refugee trauma: a preliminary case series using qigong and t’ai chi. J Altern Complement Med. 2008;14(7):801–6. 10.1089/acm.2007.0736. 18803491 10.1089/acm.2007.0736 PMC2745908

[58] Highfield ES, Lama P, Grodi MA, et al. Acupuncture and traditional Chinese medicine for survivors of torture and refugee trauma: a descriptive report. J Immigr Minor Health. 2012;14(3):433–40. 10.1007/s10903-011-9538-6. 22005843 10.1007/s10903-011-9538-6

[59] Pease M, Sollom R, Wayne P. Acupuncture for refugees with posttraumatic stress disorder: initial experiences establishing a community clinic. Explore. 2009;5(1):51–4. 10.1016/j.explore.2008.10.005. 19114264 10.1016/j.explore.2008.10.005

[60] Budd S. Supporting communities in humanitarian crises with acupuncture and integrative medicine A perspective. Med Acupunct. 2023;35(4):159–62. 10.1089/acu.2023.0012. 37928316 10.1089/acu.2023.0012 PMC10620434

[61] Askovic M, Watters AJ, Aroche J, et al. Neurofeedback as an adjunct therapy for treatment of chronic posttraumatic stress disorder related to refugee trauma and torture experiences: two case studies. Australas Psychiatry. 2017;25(4):358–63. 10.1177/1039856217715988. 28699778 10.1177/1039856217715988

[62] Askovic M, Watters AJ, Coello M, et al. Evaluation of neurofeedback for posttraumatic stress disorder related to refugee experiences using self-report and cognitive ERP measures. Clin EEG Neurosci. 2020;51(2):79–86. 10.1177/1550059419849170. 31132893 10.1177/1550059419849170

[63] Murray KE, Davidson GR, Schweitzer RD. Psychological well-being of refugees resettling in Australia. The Australian Psychological Society; 2018. Available from: https://www.psychology.org.au/assets/files/refugee-lit-review.pdf [Last accessed: 12/10/23].

[64] Ter Heide FJ, Mooren TM, van de Schoot R, et al. Eye movement desensitisation and reprocessing therapy v. stabilisation as usual for refugees: randomised controlled trial. Br J Psychiatry. 2016;209(4):311–8. 10.1192/bjp.bp.115.167775. 26892849 10.1192/bjp.bp.115.167775

[65] Kumar GS, Beeler JA, Seagle EE, et al. Long-term physical health outcomes of resettled refugee populations in the United States: a scoping review. J Immigr Minor Health. 2021;23(4):813–23. 10.1007/s10903-021-01146-2. 33515162 10.1007/s10903-021-01146-2 PMC8233239

[66] Sarris J. Herbal medicines in the treatment of psychiatric disorders: 10-year updated review. Phytother Res. 2018;32(7):1147–62. 10.1002/ptr.6055. 29575228 10.1002/ptr.6055

[67] Buchwald D, Panwala S, Hooton TM. Use of traditional health practices by Southeast Asian refugees in a primary care clinic. West J Med. 1992;156(5):507–11. 1595275 PMC1003313

[68] Foley H, Steel A, Cramer H, et al. Disclosure of complementary medicine use to medical providers: a systematic review and meta-analysis. Sci Rep. 2019;9(1):1573. 10.1038/s41598-018-38279-8. 30733573 10.1038/s41598-018-38279-8 PMC6367405

[69] Singer J, Adams JE. The place of complementary therapies in an integrated model of refugee health care: counsellors’ and refugee clients’ perspectives. J Refug Stud. 2011;24:351–75.

[70] Singer J, Adams J. Integrating complementary and alternative medicine into mainstream healthcare services: the perspectives of health service managers. BMC Complement Altern Med. 2014;14:167. 10.1186/1472-6882-14-167. 24885066 10.1186/1472-6882-14-167 PMC4048459

